# Real time MRI of border zone end-systolic regional work

**DOI:** 10.1186/1532-429X-15-S1-P191

**Published:** 2013-01-30

**Authors:** Francisco Contijoch, Walter R Witschey, Jeremy R McGarvey, Melissa M Levack, Victor A Ferrari, Norihiro Kondo, Satoshi Takebayashi, Toru Shimaoka, Chikashi Aoki, Gerald A Zsido, Joseph H Gorman, Robert C Gorman, James J Pilla

**Affiliations:** 1 University of Pennyslvania, Philadelphia, PA, USA

## Background

Heart failure results in 280,000 deaths each year in the US and post-myocardial infarction (MI) remodeling is among the leading causes. An unresolved question is the integrity of the molecular contractile apparatus in the peri-infarct or borderzone (BZ) myocardium and the resulting effect on infarct stretching and thinning and BZ expansion. A difficulty is the lack of available methods to noninvasively measure the BZ stress-strain relation under varying loading conditions in vivo. Our hypothesis is that the BZ myocardium has significantly reduced end-systolic stiffness as measured by MRI-derived regional preload recruitable stroke work (rPRSW). To test this hypothesis, we transiently varied loading conditions in post-infarct swine and measured real time LV apparent fiber length using nonlinear image reconstruction and sub-Nyquist golden angle MRI.

## Methods

Yorkshire male swine (N=3, mean weight = 60 kg) were used in an IACUC approved posterobasal MI study. At 1 week post-MI, a hydraulic balloon catheter device was positioned in the IVC. Real time golden angle radial bSSFP MRI synchronized with LV pressure during IVC occlusion (N=2) and dobutamine stress test (N=1) was performed. Images were reconstructed using Gadgetron framework (Hansen, 2012) using an iterative SENSE-based algorithm (Pruessmann, 1999) with k-space weighted imaging contrast (KWIC) temporal filtering (Song, 2000). The ventricular cavity and myocardium was segmented using a level-set algorithm (Yushkevich, 2006) and endocardial and epicardial points wall thickness (WT) was tracked using a non-rigid intensity based algorithm (Rueckert, 1999) from wall contours throughout inflow occlusion. rPRSW was quantified by the area of the Pressure- Relative Fiber Length loop.

## Results

**Figure 1:**
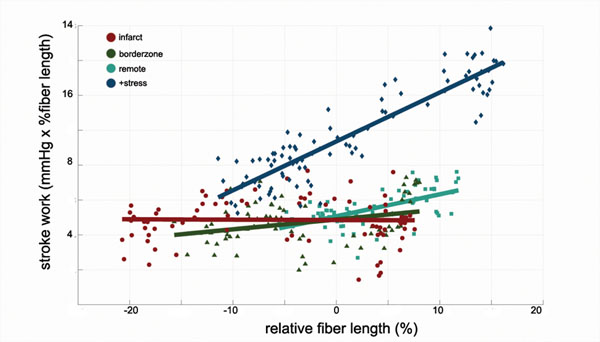


We found a decrease in relative myofiber length and stroke work in the healthy myocardium during inflow occlusion as shown in all animals. Data from one representative animal is shown in Figure 1. The slope of the lines in Fig. 1 is the regional PRSW, which was impaired in the borderzone and infarcted regions (Animal 1: rem = 26.3 mmHg, bz = 13.7 mmHg, inf = 4.3 mmHg, Animal 2: rem = 12.6 mmHg, bz = 5.7 mmHg, inf = 0.2 mmHg,. We attribute the reduction in rPRSW to decreased end-systolic stiffness in these regions 1 week post-infarction. During dobutamine infusion, the rPRSW was increased (+stress = 28.1 mmHg, baseline=12.6 mmHg), corresponding to the increase in inotropic state and increased end-systolic stiffness.

## Conclusions

Regional variations in local work performed were observed during transient preload reduction using a real time magnetic resonance imaging method. These findings provide insight about load-dependent changes in contractile function in post-infarction LV remodeling.

## Funding

R01-HL103723, R01-HL63954, R01-HL73021, and T32-EB009384.

